# AI in antimicrobial stewardship: time to involve patients?

**DOI:** 10.1017/ash.2026.10914

**Published:** 2026-10-14

**Authors:** Neda Milevska-Kostova

**Affiliations:** 1 Patients for Patient Safety Observatory, Geneva, Switzerland; 2 Radboud University Medical Center, Nijmegen, the Netherlands

## Abstract

Artificial intelligence is transforming antibiotic stewardship but largely overlooks the patient perspectives shaping prescribing decisions. Because inappropriate antibiotic use is fundamentally relational, AI must incorporate patient narratives, communication, and shared decision-making alongside clinical data. Patient-centered AI could improve prescribing, trust, adherence, and global efforts to combat antimicrobial resistance sustainably.

When artificial intelligence (AI) enters the antibiotic decision-making, it brings prediction models for infection diagnosis, antibiotic selection, and prescribing decisions. However, the stewardship tools deployed in practice are trained mostly on structured clinical and administrative data and do not systematically capture patients’ antibiotic-seeking behavior, their fears, and understanding, or needs to help them accept a decision not to prescribe. In this way, they miss an opportunity to equip healthcare providers and prescribers, especially in primary care with sufficient background on how to address patients’ concerns and communicate antibiotic (non)prescribing decisions, especially when antimicrobial therapy is not needed.

Antimicrobial resistance (AMR) is predicted to kill 10 million people annually by 2,050.^1^ Despite documented improvements of better guideline adherence,^2^ improved diagnostics, and shorter antibiotic courses,^3^ inappropriate prescribing continues. Diagnostic uncertainty, workflow pressures, and limited access to testing remain major drivers of inappropriate prescribing, but a substantial share is also, and often decisively, relational. In primary and outpatient care, patient expectations, illness narratives, implicit pressure,^4^ and the social effort of reaching shared understanding more commonly define the prescription etiquette than diagnostics or evidence-based medicine.

The data that patient involvement changes this is unambiguous. A Cochrane review found that interventions facilitating patient participation in prescribing decisions reduced antibiotic prescribing for acute respiratory infections by approximately 40%, without increasing reconsultations or reducing patient satisfaction.^5^ The most effective interventions worked not by persuading patients to accept refusal, but by eliciting concerns and making visible the trade-offs that mattered to them. When patients feel genuinely heard, they are less likely to push for antibiotics they do not need.

AI is now entering this relational space. A 2024 systematic review identified growing use of AI-based clinical decision support tools in stewardship, spanning sepsis management, urinary tract and respiratory infections.^6^ These tools have genuine potential: AI-assisted diagnostic stewardship can reduce time-to-appropriate-antibiotic and flag overuse patterns invisible to individual prescribers.

However, in many cases, the patients’ own account of what they feared, understood, and needed to feel respected and adhere to the decision is not in the AI training. Recent discussions underscore this, as patients increasingly encounter AI-generated health information outside traditional sources.^7^ Notably, evidence on AI’s impact on relational and communicative aspects of care remains largely absent. And, these relational dimensions are still important drivers of inappropriate antimicrobial prescribing and use.

Stewardship programs have long been criticized for treating patients as passive recipients^8^—managed and educated, but not genuinely involved. Patient-centered approaches have repeatedly shown that this asymmetry undermines effectiveness: patients who feel excluded from antibiotic decisions are more likely to seek advice^7^ or antibiotics elsewhere, less likely to adhere to agreed treatment plans, and less likely to trust future non-prescribing recommendations. With AI entering stewardship at speed, we have a chance to correct that asymmetry.

Addressing this gap requires more than adding patient satisfaction as an afterthought. It requires broadening AI training data to make tangible use of patient illness narratives, consultation recordings, and patient-reported outcome measures, that contain information causally relevant to prescribing behavior. Natural language processing approaches have demonstrated capacity to extract clinically meaningful signals from consultation transcripts^9^ or process patient-reported experiences;^10^ there is no technical barrier to applying these methods in stewardship contexts. Comparable nurse-facing tools already pilot AI-assisted analysis of patient-story narratives against structured care-quality indicators.^11^ Implementation could include brief structured prompts capturing patient concerns, existing patient-reported experience measures (PREMs), and other routinely collected data at the point of prescribing within current electronic health record workflows. Digital triage tools, already used to capture patient-reported symptoms ahead of a respiratory-complaint consultation, are another entry point for collecting this kind of data at scale.^12^

In addition, evaluation frameworks must be expanded. A patient who was not prescribed an antibiotic but who left the consultation confused, dissatisfied, and who re-consulted within a week represents a stewardship failure that no prescribing metric captures. AI-assisted stewardship should be evaluated on patient acceptability, reconsultation rates, and treatment adherence.

Co-designing AI tools with patients and clinicians from the outset—mapping consultation dynamics and testing interventions in situ—is more likely to be used and trusted than tools imposed on a clinical encounter without understanding its relational structure.

This matters most where the gap between AMR burden and stewardship capacity is widest. AI tools for stewardship are developed almost entirely in high-income settings and validated on populations whose illness beliefs, healthcare-seeking behaviors, and expectations around antibiotics differ markedly from those in low- and middle-income countries, where over-the-counter antibiotic access is routine and the social meaning of leaving a consultation without medication is entirely different. If these tools are not built to account for that, they will deepen rather than close the global stewardship divide. The same divide runs within countries: those with least access to antibiotics and care often have least access to digital tools, and patient-centered AI must not end up serving only those already best served.

This relational gap is not confined to clinician-facing tools; it recurs, more consequentially, where AI mediates antibiotic decisions directly with patients, with no clinician in the loop. The scale of the problem is not hypothetical. Practices of antimicrobial self-medication and leftover sharing persist across LMICs: pooled estimates from LMIC-focused reviews range from roughly one-third to over three-quarters of adults, with antibiotics typically obtained from pharmacies and informal retail outlets without any prescription, diagnosis, or clinical oversight.^13,14^ These are the same settings that carry the highest AMR-attributable mortality globally.^1^ Into this landscape, AI is arriving not as a governed clinical tool but as an unregulated, freely available source of diagnostic and treatment advice. Generative AI chatbots are now used by many people for health guidance, and emerging evidence already links their use to self-diagnosis and antibiotic self-prescription, including among populations where informal antibiotic access is already common.^15^ Crucially, regulatory oversight of these consumer-facing tools lags furthest behind where antibiotic regulation is weakest, leaving a compounding gap of unsupervised or clinically justified antimicrobial use. Without scientific and policy attention to this convergence, penetration of AI for wide use brings risks of actively accelerating self-medication-driven AMR in the countries least able to absorb its consequences.

Inappropriate antibiotic use is, at its heart, a relational problem, and AI has the potential to transform that relational space. Existing evidence speaks of positive effects of human-facilitated shared decision-making in antibiotic use; whether using AI’s potential by incorporating patient-generated data to reproduce—or even amplify—this effect is an open, empirically untested question that stewardship research should prioritize.

## Data Availability

Not applicable.
